# Molpher: a software framework for systematic chemical space exploration

**DOI:** 10.1186/1758-2946-6-7

**Published:** 2014-03-21

**Authors:** David Hoksza, Petr Škoda, Milan Voršilák, Daniel Svozil

**Affiliations:** 1 Laboratory of Informatics and Chemistry, Faculty of Chemical Technology, Institute of Chemical Technology Prague, Technická 5, CZ-166 28 Prague, Czech Republic; 2 SIRET Research Group, Department of Software Engineering, Faculty of Mathematics and Physics, Charles University in Prague, Malostranské nám. 25, CZ-118 00 Prague, Czech Republic; 3 Institute of Molecular Genetics, Academy of Sciences of the Czech Republic, Vídeňská 1083, CZ-142 20 Prague, Czech Republic

**Keywords:** Chemical space exploration, De-novo design, Lead discovery, Structure generation, In silico ligand design, Chemical biology tools

## Abstract

**Background:**

Chemical space is virtual space occupied by all chemically meaningful organic compounds. It is an important concept in contemporary chemoinformatics research, and its systematic exploration is vital to the discovery of either novel drugs or new tools for chemical biology.

**Results:**

In this paper, we describe Molpher, an open-source framework for the systematic exploration of chemical space. Through a process we term ‘molecular morphing’, Molpher produces a path of structurally-related compounds. This path is generated by the iterative application of so-called ‘morphing operators’ that represent simple structural changes, such as the addition or removal of an atom or a bond. Molpher incorporates an optimized parallel exploration algorithm, compound logging and a two-dimensional visualization of the exploration process. Its feature set can be easily extended by implementing additional morphing operators, chemical fingerprints, similarity measures and visualization methods. Molpher not only offers an intuitive graphical user interface, but also can be run in batch mode. This enables users to easily incorporate molecular morphing into their existing drug discovery pipelines.

**Conclusions:**

Molpher is an open-source software framework for the design of virtual chemical libraries focused on a particular mechanistic class of compounds. These libraries, represented by a morphing path and its surroundings, provide valuable starting data for future *in silico* and *in vitro* experiments. Molpher is highly extensible and can be easily incorporated into any existing computational drug design pipeline.

## Background

Chemical space is populated by all chemically meaningful and stable organic compounds [1-3]. It is an important concept in contemporary chemoinformatics research [4,5], and its exploration leads to the discovery of either novel drugs [2] or new tools for chemical biology [6,7]. It is agreed that chemical space is huge, but no accurate approximation of its size exists. Even if only drug-like molecules are taken into account, size estimates vary [8] between 10^23^[9] and 10^100^[10] compounds. However, smaller numbers have also been reported. For example, based on the growth of a number of organic compounds in chemical databases, Drew *et al.*[11] deduced the size of chemical space to be 3.4 × 10^9^. By assigning all possible combinations of atomic species to the same three-dimensional geometry, Ogata *et al*. [12] estimated the size of chemical space to be between 10^8^ and  10^19^. Also, by analyzing known organic substituents, the size of accessible chemical space was assessed as between 10^20^ and 10^24^[9].

Such estimates have been put into context by Reymond *et al.*, who produced all molecules that can exist up to a certain number of heavy atoms in their Chemical Universe Databases: GDB-11 [13,14] (2.64 × 10^7^ molecules with up to 11 heavy atoms); GDB-13 [15] (9.7 × 10^8^ molecules with up to 13 heavy atoms); and GDB-17 [16] (1.7 × 10^11^ compounds with up to 17 heavy atoms). The GDB-17 database was then used to approximate the number of possible drug-like molecules as 10^33^[8].

While virtual chemical space is very large, only a small fraction of it has been reported in actual chemical databases so far. For example, PubChem contains data for 49.1 million chemical compounds [17] and Chemical Abstracts consists of over 84.3 million organic and inorganic substances [18] (numbers as of 12. 3. 2014). Thus, the navigation of chemical space is a very important area of chemoinformatics research [19,20]. Because chemical space is usually defined using various sets of descriptors [21], a major problem is the lack of invariance of chemical space [22,23]. Depending on the descriptors and distance measures used [24], different chemical spaces show different compound distributions. Unfortunately, no generally applicable representation of invariant chemical space has yet been reported [25].

Approaches to chemical space navigation can be categorized by the way in which molecular structure and properties are encoded [26]. The two main methods used are descriptor vectors and graphs. In descriptor-based chemical space, molecules are treated as multidimensional vectors consisting of molecular descriptors [21]. To analyze such multidimensional data, dimensionality reduction mapping techniques are used [27], mainly Principal Component Analysis (PCA) [28] and/or Multidimensional Scaling (MDS) [29]. For example, the chemical global positioning system ChemGPS [30] utilizes PCA to create a ‘navigation map’ in drug-like space. Another PCA-based system suitable for the comparison of chemical libraries is Delimited Reference Chemical Subspace (DRCS) [31,32].

In graph-based chemical space, compounds are typically simplified into their molecular scaffolds [33]. In the scaffold tree algorithm [34], large molecular data sets are organized into a unique tree hierarchy by iterative removal of rings from more complex scaffolds. The scaffold tree has been successfully applied to the analysis of chemical data [35,36] and to the identification of new bioactive regions in chemical space [37]. Other scaffold-capturing organization schemes include the molecular equivalence number classification system [38], the related chemotype approach [39] and hierarchical scaffold clustering (HierS) [40].

The computer-assisted *de novo* design of bioactive molecules is another important way of navigating chemical space. *De novo* design requires the assembly of candidate compounds, which are then used to search for novel structures [41]. Because ‘combinatorial explosion’ [42] makes it impossible to consider all theoretically conceivable compounds, this search space must first be reduced by incorporating as much chemical knowledge as possible. Two major approaches exist for assembling candidate compounds: atom-based and fragment-based. While atom-based techniques require molecules to be built atom by atom [43], in fragment-based approaches molecules are formed from predefined molecular building blocks [44]. The key element in fragment-based assembly is an adaptive scheme for virtual molecular evolution [45]. However, despite its advantages, fragment-based molecular structure evolution programs do not follow a structural continuum [46]. Because of this, less coarse-grained approaches have been proposed. The median molecules approach [47,48] generates structures similar to two different starting compounds by a graph-based genetic algorithm. This algorithm uses multiobjective optimization that applies a Pareto ranking scheme to the evolution of candidate solutions. Molecule Evaluator [49] uses crossover and mutation operators to evolve a set of molecules, the quality of which is assessed by the user. Bishop *et al.* developed [50] an approach that uses chemical reactions as structural mutations. van Duersen *et al.*[46] represented chemical space as a graph with molecules as nodes and structural mutations as edges. Their SPACESHIP program generates a structural continuum between two molecules by the iterative application of mutation and selection cycles. Yu devised a molecular enumerator that produces a diverse set of natural-product-like [51] or drug-like [52] molecules by attaching randomly selected fragments to the molecular core. Algorithm for Chemical Space Exploration with Stochastic Search (ACSESS) combines molecular evolution and maximum diversity methods to create libraries representative of various chemical spaces [53].

For the systematic discovery of chemical space, we proposed a method of ‘molecular morphing’ [54]. Our method is inspired by the morphing effect used in animation films, in which one image morphs into another through seamless transition. Similarly, a start molecule is converted into a target molecule by the application of morphing operators that correspond to simple structural changes, such as the addition or removal of an atom or a bond. If the start and target molecules belong to the same mechanistic class of compounds (i.e. they are active at the same receptor), the molecules encountered along the morphing path and within its surroundings represent a focused virtual library. Such a library provides valuable starting data for subsequent *in silico* experiments that aim to identify more potentially active leads. The predicted leads can be further optimized and their biological activity subsequently assessed in *in vivo* experiments.

Molpher is a freely-available client–server application that includes the following features: a user-friendly graphical interface; a wide range of molecular representations and similarity measures; the interactive modification of the algorithm’s parameters; the visualization and inspection of explored space; and the export of generated structures. In addition, Molpher is designed to be used as a software framework that can easily incorporate new molecular representations, similarity measures and visualization techniques.

## Implementation

Molpher is written in C++, is open source and can be freely downloaded (GNU Public License v3). For standard tasks, it uses Boost C++ Libraries [55]. Chemical functionality is provided by the open source chemoinformatics toolkit RDKit [56], which offers a reasonable level of thread-safety, a native C++ application programming interface, and a vast number of fingerprints and similarity coefficients. Molpher leverages the computational power of modern CPUs by dividing chemical space exploration between individual CPU cores. This parallelization is implemented by employing the Intel® Thread Building Block Library (TBB) [57].

### Algorithm

In this section, we briefly describe Molpher’s molecular morphing algorithm. A more detailed description of the algorithm and its parameters is given in Additional file 1.

**Figure 1:**
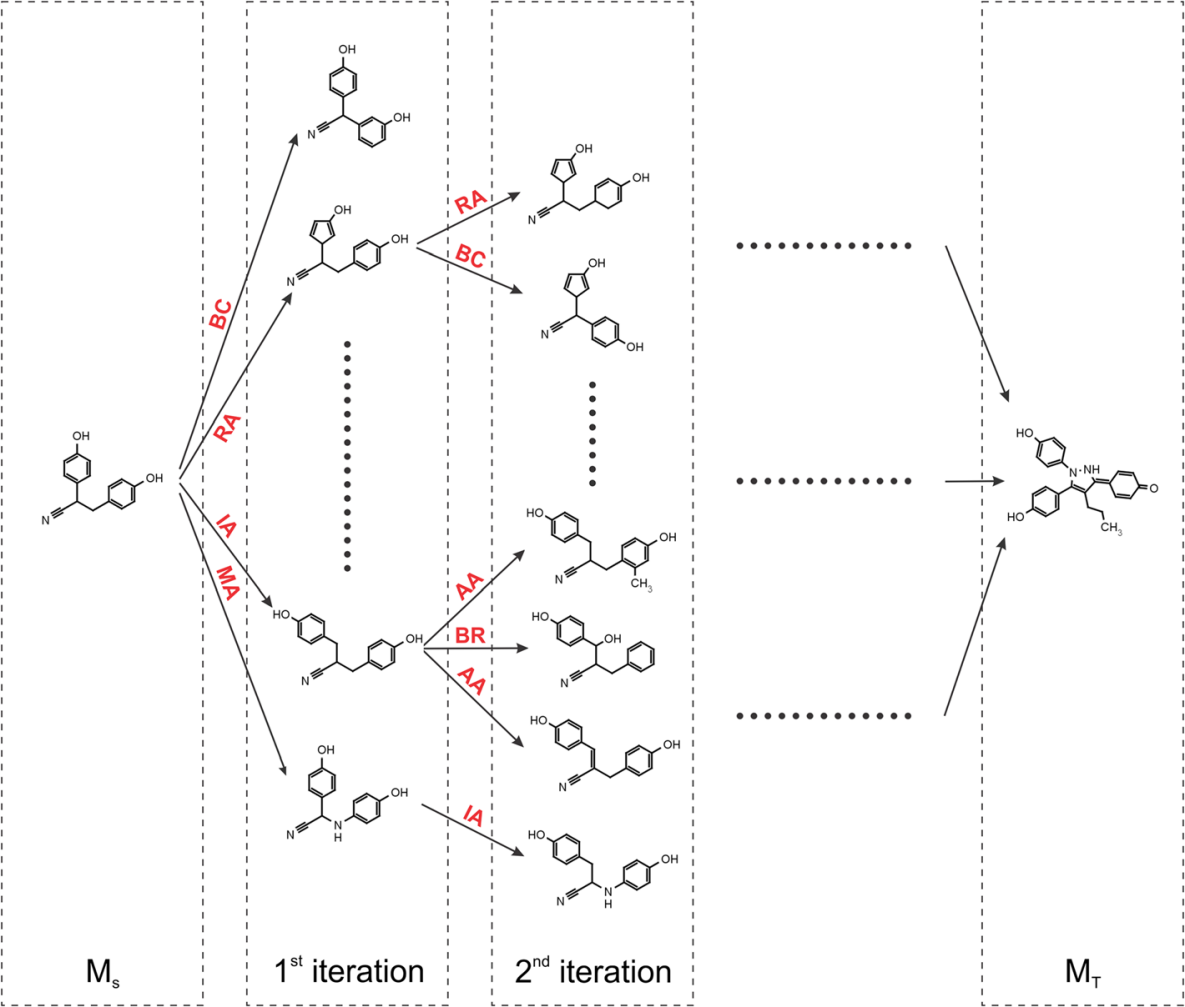
**Principles of the molecular morphing approach.** Molecular morphing generates a path in chemical space consisting of structures (referred to as morphs) lying between a start molecule ***M***_***S***_ and a target molecule ***M***_***T***_. In each iteration ***i*** + 1 a set of morphs is generated by randomly applying morphing operators (see Figure 2) at molecules from the iteration ***i***. The morphs from the iteration ***i*** are accepted as candidates for the iteration ***i*** + 1 with the probability derived from their distance to the target molecule. A more detailed description of the algorithm is given in Additional file 1.

**Figure 2:**
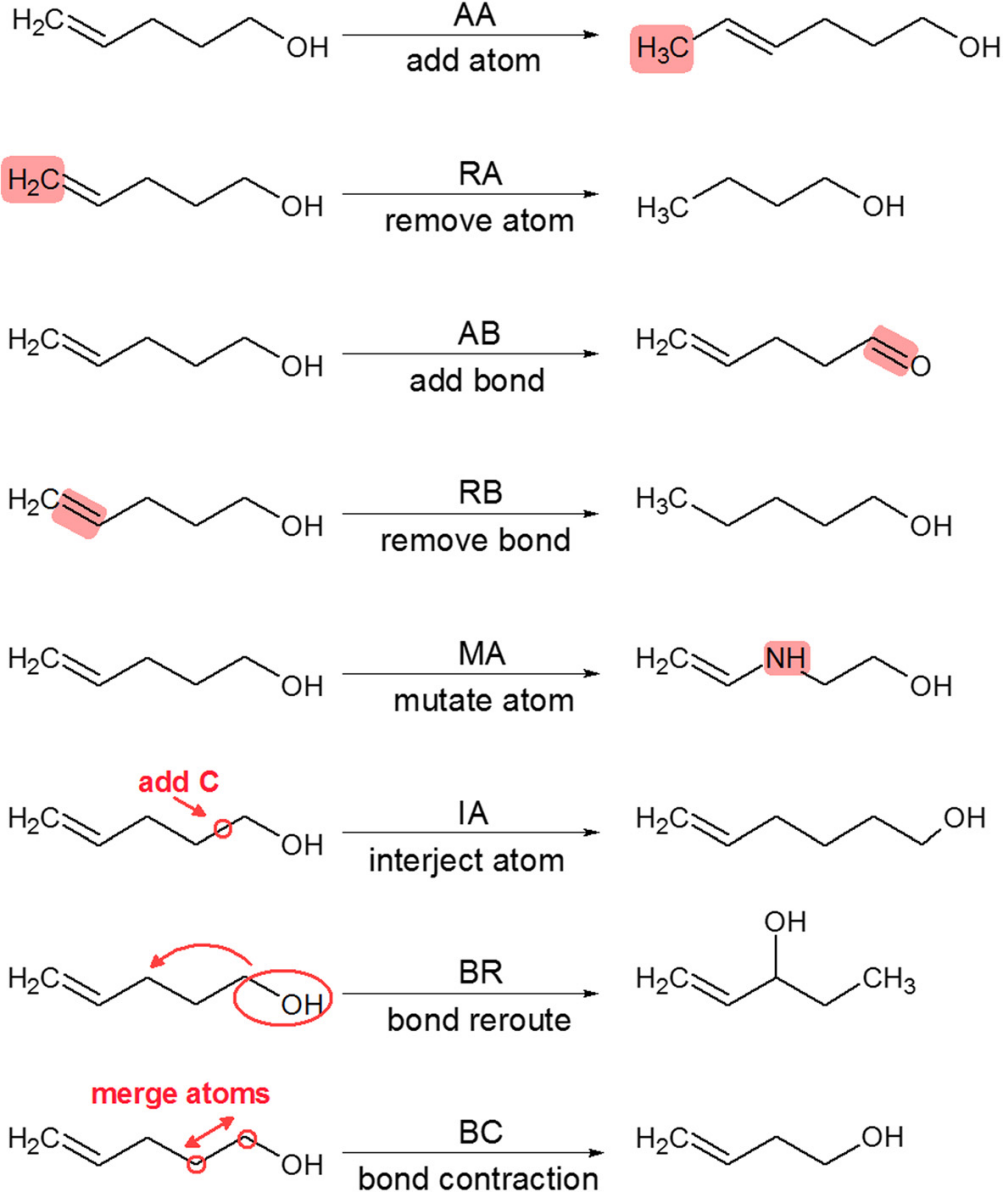
**Morphing operators.** Morphing operators represent simple structural changes (mutations) that lead to the transformation of the ‘reactant’ molecule (left) to the ‘product’ molecule (right).

The main task of molecular morphing is to find a path in chemical space between a start molecule *M*_*S*_ and a target molecule *M*_*T*_. The principles of our algorithm for doing this are illustrated in Figure 1. This algorithm is based on an iterative process where, in each iteration *i* + 1, a set of molecules (referred to as morphs) *M*_*i* + 1_ is generated from the previous set *M*_*i*_. The morphs are formed by the application of randomly selected morphing operators (see Figure 2) acting at randomly selected molecular positions. Each morph formed must comply with basic valency rules. The resulting morphs can be filtered by their molecular weight or by their synthetic accessibility, SAScore [58]. Once filtered, they are sorted by their distance from *M*_*T*_. This produces a list, from which the required number of morphs, *M*_*i* + 1_, is selected. Some of the molecules from *M*_*i*_ may not generate any offspring during the *i* + 1 iteration. If this happens over several iterations, these molecules are discarded from the exploration process. The process ends when the target molecule is located; namely, when one of the morphs is identical with the *M*_*T*_. The sequence of morphs from *M*_*S*_  to *M*_*T*_ defines the path in chemical space. The runtime required for path identification can be limited to a specific number of iterations, in which case the solution is partial.

### Software architecture

Molpher is a client–server application where data intensive tasks, such as chemical space exploration, are delegated to the server. The client graphical user interface, developed using the Qt library [59], provides the only means of changing the server settings. From the client, the user can create and manage jobs, change their settings and display their results. The client–server architecture also enables the exploration process to be divided among multiple clients, any of which can be disconnected from a running job and, if necessary, later reconnected. Both client and server can reside on the same machine or be used as separate components designed to communicate over a network. The server is a command line application that listens for client connections on a specific port. Whenever new results are available, the server broadcasts them to all connected clients. There is no ‘master client’ instance with exclusive rights to control the server; all clients are equal. Any client can create jobs (exploration tasks) on the server and adjust the properties of the currently running jobs. Jobs can be password protected to prevent other clients from modifying them. In addition, the server can be run in batch mode, in which it behaves as a non-interactive program with jobs passed as command line arguments. After performing the specified jobs and storing their results, the server terminates. With its overall functionality, Molpher users can easily incorporate molecular morphing into their drug discovery pipelines.

### Graphical user interface and its capabilities

In the following paragraphs, we briefly describe the graphical user interface (GUI) of the Molpher client. For a more detailed description, the reader is referred to the Molpher User manual [60].

**Figure 3:**
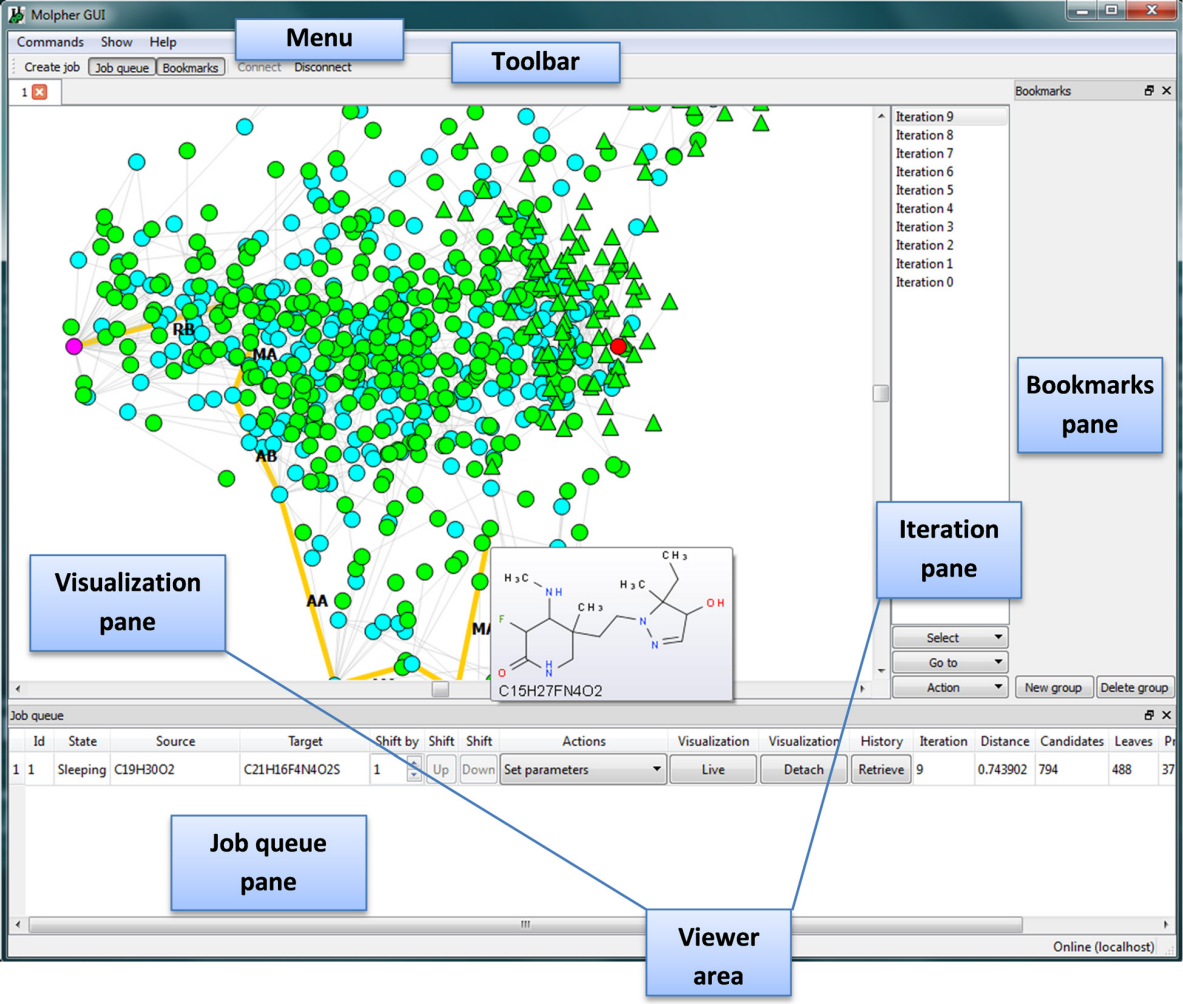
**Graphical user interface of the Molpher client.** Display of the *Job queue* and *Bookmarks* panes can be turned off from the detachable toolbar. *Viewer area* consists of the *Visualization* and *Iteration* panes.

**Figure 4:**
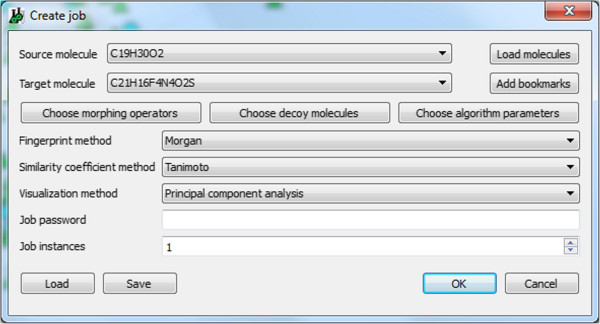
***Create job *** **dialog box.**

Molpher’s main window consists of a menu, detachable toolbar, *Bookmarks* pane, *Jobs queue* pane and viewer area (see Figure 3). The viewer area is subdivided into *Visualization* and *Iteration* panes. The user starts an exploration task by clicking the *Create job* button, which invokes the *Create job* dialog box (see Figure 4). In this dialog box, the user can specify a start/target molecule pair and one or more of the following parameters: fingerprinting method, similarity coefficient method, visualization method, set of morphing operators (see Figure 2) and decoy set (see later). Both start and target molecules must be imported from an external SDF file. They may be imported from different files or from a single file; in the latter case, the user must select the start/target molecule pair from the drop-down menu.

The molecular morphing algorithm is controlled by parameters accessed by clicking the *Choose algorithm parameters* button (see Additional file 1). The two most important parameters influencing the exploration process are *Fingerprint representation* and *Similarity coefficient*. Morgan fingerprints, which belong to the family of circular fingerprints [61], and the Tanimoto coefficient are set as defaults because, in our experience, they offer reasonable performance in the vast majority of cases. However, many other fingerprints and similarity coefficients are implemented (see Additional file 2). Also, it is possible to limit a morph’s molecular weight (default is 500 Da) so that the algorithm does not produce overly complex molecules. From the same dialog, a prediction of the morph’s synthetic feasibility [58] can be switched off. When the parameters have been setup, the client’s configuration can be saved in an SNP (snapshot) file and restored as required by using the *Save* and *Load* buttons, respectively, in the *Create job* dialog box.

Typically, Molpher explores relatively direct paths between the start/target molecule pair, but the user may request the process to explore remoter areas of chemical space. This is done by defining additional ‘decoy molecules’ via the *Create job* dialog box. The presence of these decoys modifies the calculation of the morph’s distance to the target molecule. In this case, the program averages two distances: the distance of the morph to the target molecule, and the distance of the morph to the decoy molecule. In these calculations, only the distance to the closest decoy is considered.

The user can also specify molecules referred to as bookmarks in the *Create job* dialog box. Bookmarks can be shared between individual jobs during a client session. Bookmarked molecules can be made available for reuse in later sessions by being stored in a user defined group and exported into an SDF file. Bookmarks are most useful when a job is having difficulty in identifying the path. In such a case, it can be helpful to setup a differently configured job using the same start and target molecules. The user can then bookmark promising candidates in the original job and load them as decoys into the new job, thereby facilitating faster algorithm convergence.

A morphing process can be inspected in the *Visualization* pane (see Figure 3). The visualization of chemical space depends on both the molecular representation and on the visualization technique used to reduce a multidimensional space to two or three dimensions [27,62]. Currently, two visualization techniques are implemented in Molpher: Principal Component Analysis (PCA), a linear dimension reduction method, and Kamada-Kawai, a graph-layout-based method. The visualization method is selected in the *Create job* dialog box and may be changed later, even for a running job. Each new iteration, the visualization is recalculated using all present morphs. Because the visualization method has such an impact on the user’s experience, Molpher’s modular architecture enables users to implement additional visualization techniques.

PCA transforms correlated variables into uncorrelated ones [63]. The uncorrelated variables, termed principal components, are constructed as linear combinations of the original variables. The dimension of the original data can be reduced by retaining only a small number of principal components that describe the predefined amount of variability. In Molpher, PCA is used to reduce the original chemical space to two dimensions.

Morphing space can also be considered as a graph, in which each pair of nodes represents two molecules separated by a single morphing operator, which is assigned to the connecting edge. Several graph-based layout algorithms exist for the visualization of graphs in an aesthetically pleasing way [64]. In Molpher, we implemented the force-directed-based Kamada-Kawai (KK) method [65]. Using this method, nodes are positioned in 2D space so that the number of edge crossings is minimized, and both nodes and edges are distributed uniformly. In KK, every pair of nodes is assigned a value *d*_*ij*_ that corresponds to the shortest path between these nodes. However, in Molpher *d*_*ij*_ corresponds to the structural similarity between morphs. In addition, each node pair is also characterized by its Euclidean distance in 2D space. Each layout is characterized by its energy *E*, which is derived from the difference between the *d*_*ij*_ and Euclidean distances of all node pairs. The KK algorithm iteratively generates a layout with the lowest value of *E*.

When the job is created, it is immediately run, receiving a unique ID number. The job can be checked in the *Job queue* (see Figure 3), in which it will appear in one of four possible statuses: *Running*, *Live*, *Sleeping* and *Finished. Live* status means that the job is waiting in a queue and is scheduled to run as soon as free resources appear. Both *Running* and *Live* jobs can be put into *Sleeping* mode by selecting *Set parameters* from the *Action* column of a job’s entry in the *Job queue*. In addition, the *Action* column (see Figure 3) enables the user to make on-the-fly modifications to the algorithm’s parameters, as well as to select morphing operators, fingerprints, similarity coefficients and visualization methods.

Three different tabs can be used to visualize a job:

1. The *Live* tab is invoked by clicking the *Live* button in the *Job queue* for the relevant job. Visualization in this tab is refreshed after each iteration. For each job, only one *Live* tab can be opened at a time. The *Live* tab is indicated in the tab caption by the job’s ID (e.g. ‘1’).

2. The *Detached* tab is invoked by clicking the *Visualization-Detach* button in the *Job queue* for the relevant job. This displays a graphical snapshot of the job at the time at which the tab is opened. There is no limit to how many of these tabs can be detached from a particular job. The *Detached* tab is indicated in the tab caption by backslash followed by the job’s ID (e.g. ‘\1’).

3. The *Adhoc* tab is invoked by double-clicking a job iteration in the *Iteration* pane in the *Job queue* (see Figure 3). This tab can display multiple snapshots of iterations from different jobs at any one time. The *Adhoc* tab is indicated in the tab caption by the job’s ID and iteration number separated by a colon (e.g. ‘1 : 51’ means the 51^st^ iteration of job 1).

**Figure 5:**
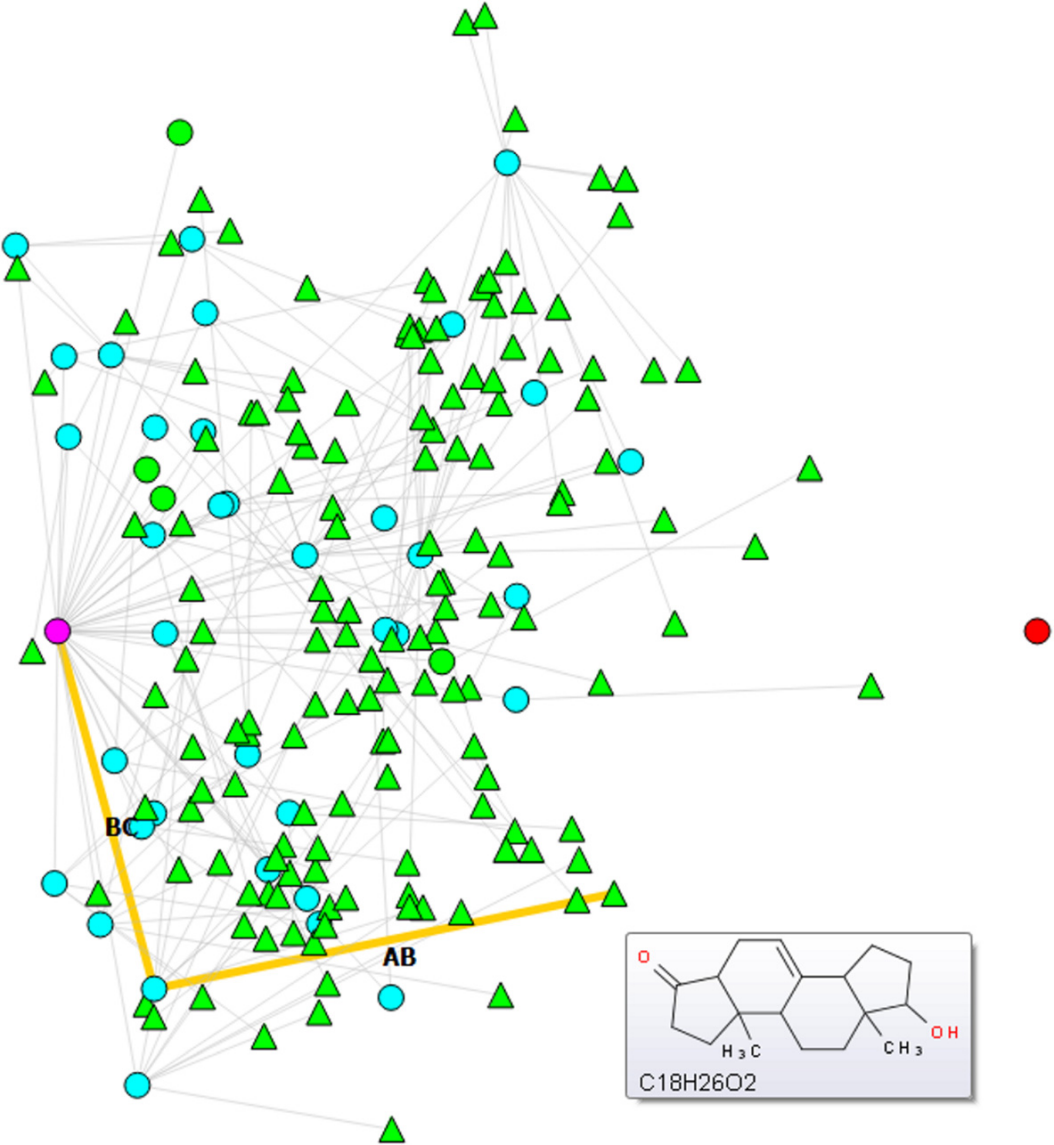
**Depiction of a structure in Molpher.** An orange line is the highlighted path from the start molecule to the particular morph.

Molecules in the explored space are represented using the following colored symbols: a start molecule as a magenta circle; a target molecule as a red circle; an inner tree node as a cyan circle; a tree leaf as a green circle; a decoy as an orange circle; and a new morph in the actual iteration as a green triangle (see Figure 3). A list of these symbols and what they signify is available by pressing the F1 key. If the user hovers the mouse over any of these symbols, the corresponding molecular structure is depicted (see Figure 5) by the external utility indigo-depict, which is part of the Indigo Toolkit [66].

Holding the left mouse button and dragging enables the user to select only the required area of chemical space. Morphs can be added or removed from the selection by clicking the left mouse button while holding the Ctrl key. More specific selections, such as selecting all new candidates or all inner tree leaves, can be made via the *Select* button from the *Iteration* pane. Among other things, selected morphs can be exported into an SDF file.

Clicking the middle mouse button on any node highlights the path from the start molecule to the selected morph (see Figure 5). The user can then inspect all morphs lying on this path. The user can move the whole view by holding the right mouse button and zoom it by turning the mouse wheel.

Clicking the right mouse button on any node invokes a context menu, from which the active morph can be bookmarked. In addition, molecules lying on the path between the active morph and the start molecule can be selected, and the whole path easily exported into an SDF file. The active morph can also be copied onto a clipboard as a SMILES string or as a summary formula. When Marvin suite from ChemAxon [67] is installed, the active morph can be opened externally in Marvin Sketch, Marvin Space or Marvin View. The last two items in the context menu enable the user to perform either an exact match search or a similarity search in the Pubchem [68], ZINC [69] or ChEMBL [70] databases.

**Figure 6:**
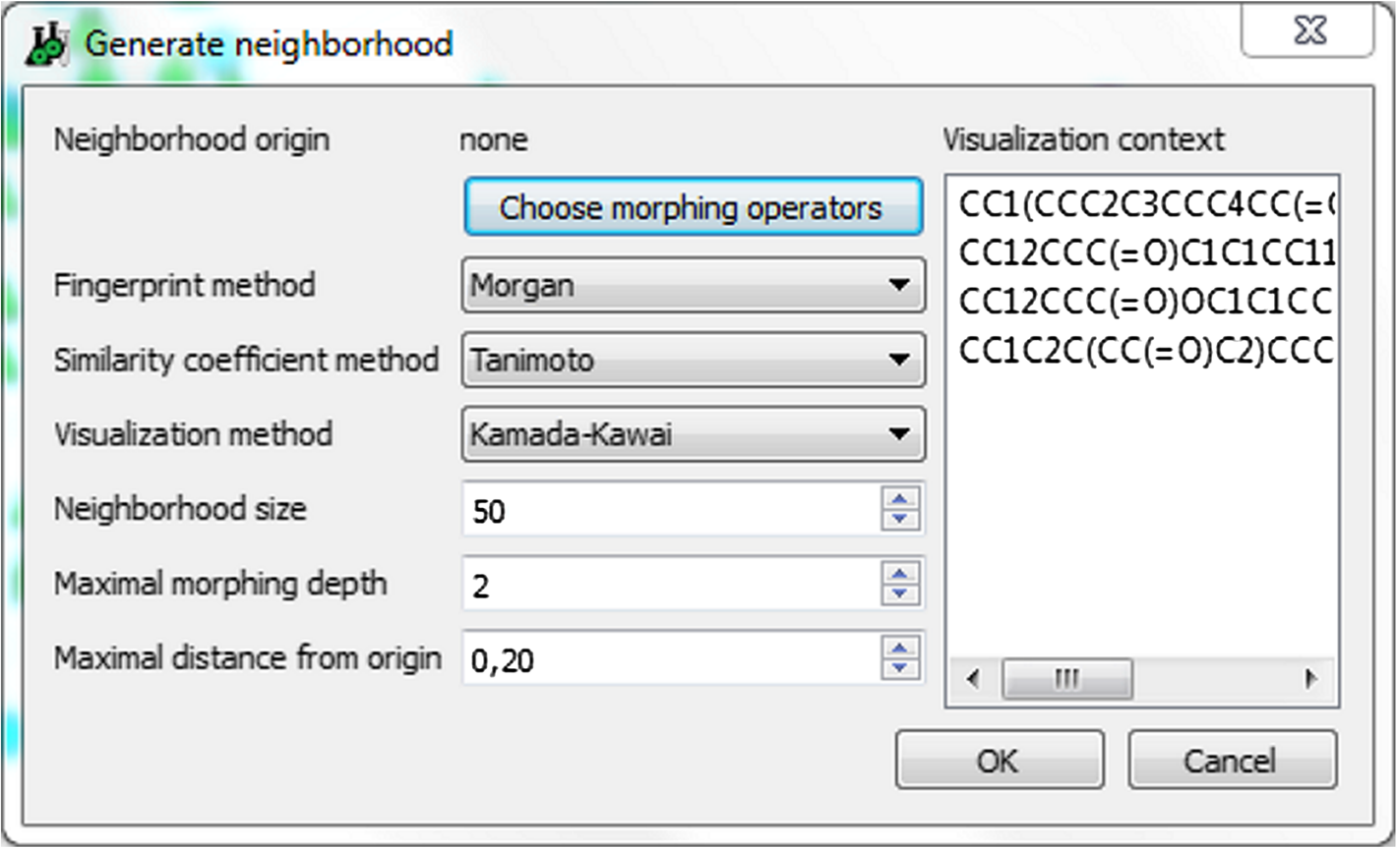
***Generate neighborhood *** **settings.**

Another rich set of features is available on the *Action* menu, which is accessed by clicking the *Action* button on the *Iteration* pane. To expand chemical space coverage in the vicinity of a potentially interesting molecule, its near neighbourhood can be explored. A neighbourhood is defined by its origin molecule, by its maximum size (i.e., the maximum number of neighbour molecules), by its maximum radius given as a similarity coefficient distance, and by its maximum depth in terms of the number of morphing operations (see Figure 6). A neighbourhood can be visualized by right-clicking the origin molecule in the *Visualizatio*n pane and by selecting *Toggle neighborhood origin* from the menu. In the visualization, identified neighbours will be placed at positions given by the so-called visualization context (VC). The VC contains molecules selected by dragging the left mouse button (or by Ctrl + left mouse button) in the *Visualization* pane. If no molecules are selected, the VC remains empty and neighbours are placed in the 2D visualization at random positions. When VC is not empty, the visualization is calculated from the VC and the identified neighbourhood molecules. To position neighbours with respect to the whole exploration tree, all molecules must be dragged into the VC.

**Figure 7:**
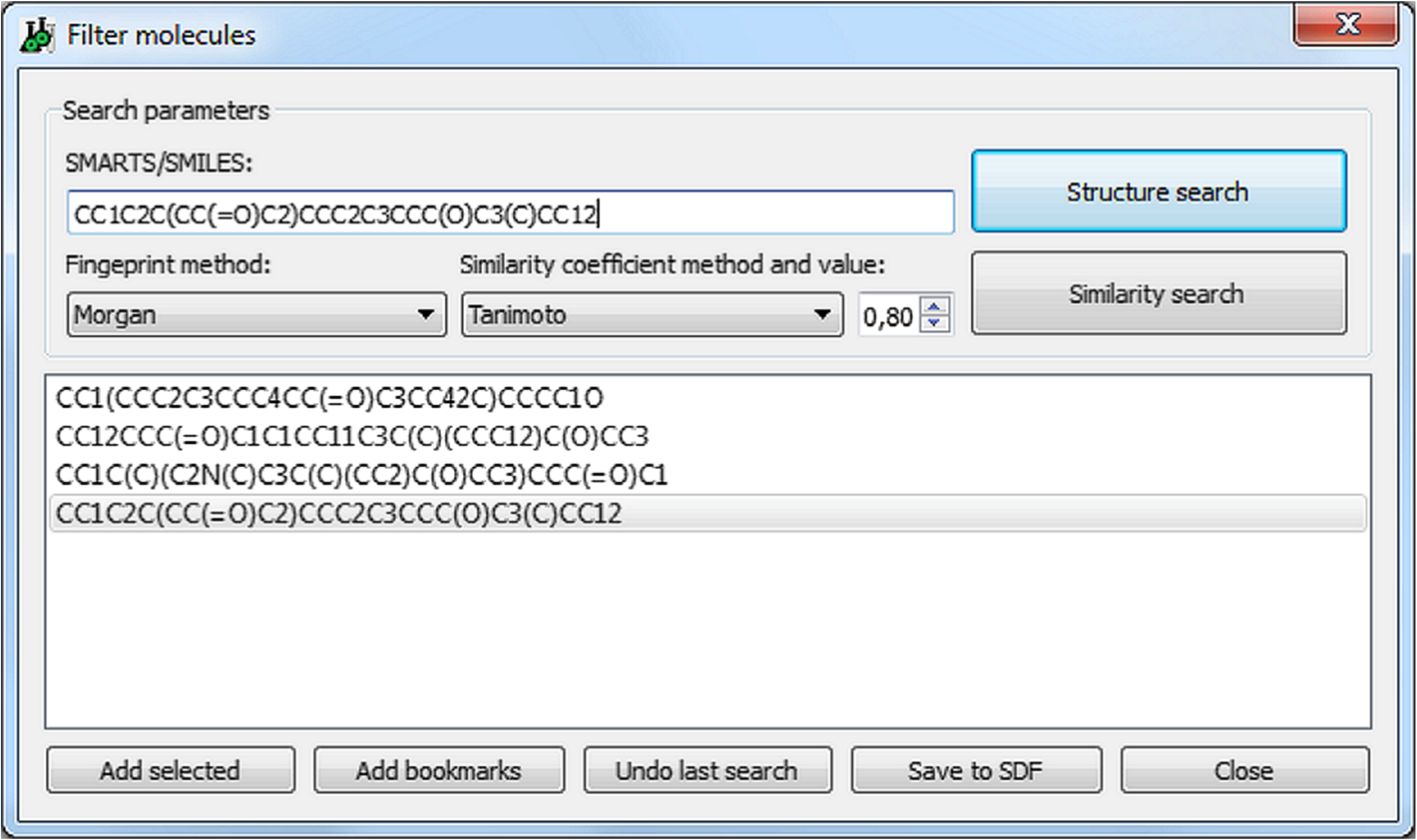
Filter molecules dialog.

The set of selected molecules can be further limited by identifying those with specific or similar structures. This feature is available by clicking the *Action* button on the *Iteration* pane and selecting *Filter molecules* (see Figure 7). The user can query using either SMILES [71] or SMARTS [72] strings. Selected molecules can be bookmarked for further use by invoking *Create molecule bookmarks* from the *Action* menu.

The final item in the *Action* menu, *Pubchem*, enables the Pubchem database to be searched for an exact match or for neighbourhood generation. Neighbourhood molecules are depicted as yellow circles. If an exact match is found in Pubchem for any of the selected molecules, that molecule changes its shape to a rhombus.

## Results and discussion

Molpher is designed to propose new candidates for biological testing by the controlled exploration of chemical space. A focused chemical library is represented by a morphing path and its surroundings. To assess the ability of Molpher to find such a path, we selected three sets of start/target molecule pairs from the PubChem database [68] (all structures can be found in Additional file 3). These sets differed only in terms of their similarities, which were evaluated using the PubChem fingerprint structural key [73] and Tanimoto similarity coefficient. Each set consisted of 20 start/target pairs. Molecules in the *D1* set shared 70-80% similarity; molecules in the *D2* set 50-60% similarity; and molecules in the *D3* set 30-40% similarity. To test Molpher’s speed, we used a machine with 4 Intel® Xeon® E5450 3GHz processors running Windows Server 2008 R2. We restricted the experiments to a single CPU thread and limited the exploration process to 1000 iterations. To perform additional computations and further fine tune Molpher’s parameters, we also used the Czech National Grid Infrastructure, MetaCentrum. To accommodate the non-deterministic character of molecular morphing, we ran each start/target exploration five times using the default Morgan fingerprint and Tanimoto distance settings. The molecular weight of morphs was limited to 500 Daltons. Each exploration was run five times with the synthetic feasibility filter turned on and five times with it turned off.

**Figure 8:**
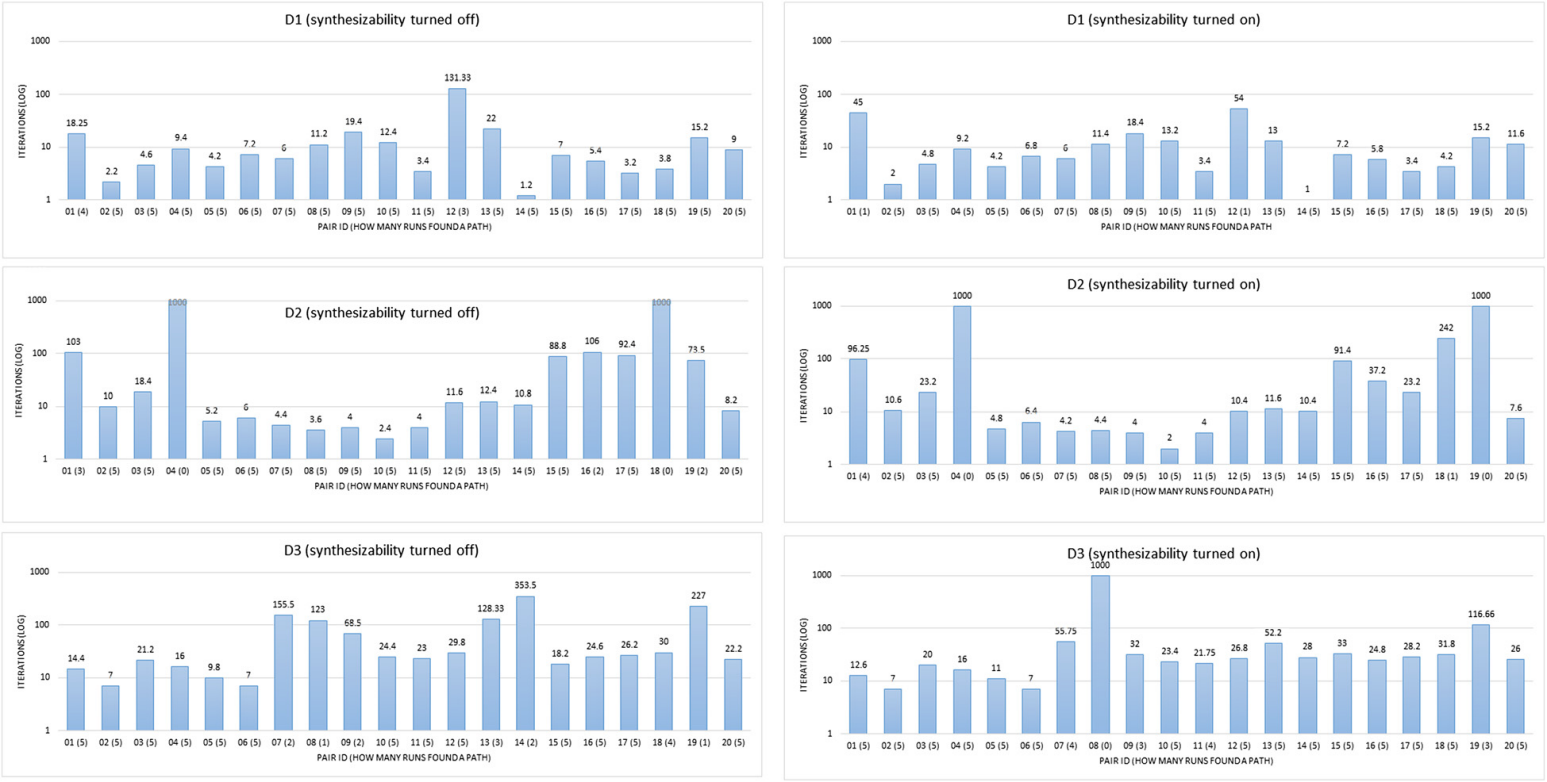
**Ability of Molpher to find a path in chemical space.** Each set (*D1*, *D2*, *D3*) contains 20 start/target molecule pairs. Molecular morphing for each start/target pair was run 5 times. The number of iterations was limited to 1000 for each run. A result for each start/target molecule pair is shown as a bar with height reflecting an average number of iterations in successfully finished jobs (i.e., jobs in which the path was identified before reaching the upper limit of 1000 iterations). Each bar is annotated by the start/target pair’s numerical ID followed by the number of successfully finished runs given in parentheses. If the bar’s height equals to 1000 neither of five exploration tasks was finished within the limit of 1000 iterations. Exploration was run with synthetic feasibility filter turned off (graphs on the left) and on (graphs on the right).

Figure 8 shows Molpher’s ability to find a path for the *D1*, *D2* and *D3* datasets. Each bar represents the average number of iterations needed to find the path between the given start/target molecule pair. This average was computed over runs in which the path was found in less than 1000 iterations. If during any individual run the path was not found, the value for that run was set to 1000.

As expected, the number of iterations increased as the similarity decreased between the start/target structures (see Table 1). In some cases, when the synthetic accessibility filter was on, the average number of iterations decreased (e.g., pair 20 in the *D1* dataset, see Figure 8) and the number of successfully completed runs increased (e.g., pair 7 in *D3* dataset, see Figure 8). However, in a few cases (pair 1 in the *D1* dataset or pair 19 in the *D2* dataset, see Figure 8), having the filter on led to a slight deterioration in the exploration process. Thus, we conclude that restricting the chemical space explored to only those molecules amenable to chemical synthesis does not significantly influence the ability of Molpher to find the path (see Table 1).

**Table 1:** Median number of iterations needed to generate the path

**Dataset**	**Synthesizability off**	**Synthesizability on**
*D1*	6.5	6.6
*D2*	10.4	10.5
*D3*	23.8	25.4

We found that the runtime of the algorithm is influenced by the following factors: the similarity of the start/target molecule pair; the settings of parameters (primarily similarity coefficient and molecular fingerprint); and the hardware on which the calculation is run. Using the default parameters (Tanimoto coefficient and circular ECFP-like Morgan fingerprint), the path was generated in 9.5 minutes on average (averaged over all start/target pairs from all datasets) using a single processor core. However, when Molpher was run on multiple cores, every path was identified within 5 minutes. Such speeds makes Molpher highly suitable for data intensive tasks.

**Figure 9:**
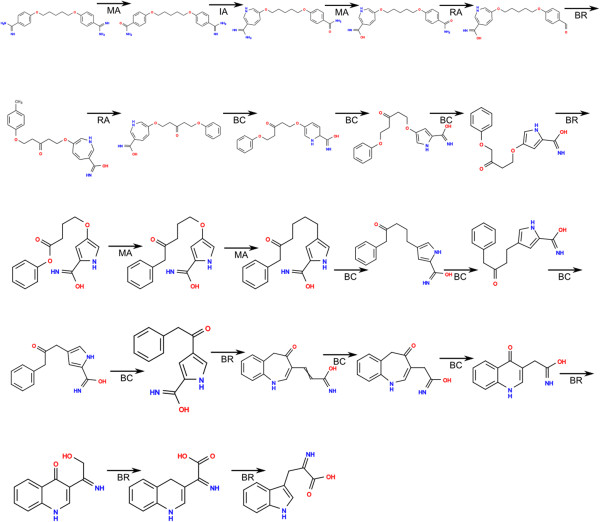
**An example of the path between pentamidine (CID 4735) and 2-imino-3-(1H-indol-3-yl)propanoic acid (CID 5599) from dataset *D3*.** The path was generated using Morgan fingerprint, Tanimoto coefficient, and synthetic accessibility filter turned on. The arrows’ labels show the used morphing operators (see Figure 2). The depiction of the path was done by OpenBabel [74].

Figure 9 displays an example of the path for molecular pair 17 [pentamidine (CID 4735) and 2-imino-3-(1H-indol-3-yl)propanoic acid (CID 5599)] from *D3*. Additional file 4 shows all five paths for this pair. Close inspection of these paths reveals that the operators used did not increase the size or complexity of the morphs. Specifically, many bond contraction (BC) and remove atom (RA) operations were used, together with operations that preserved the size of the molecule and only modified the atom or bond types [mutate atom (MA) and bond reroute (BR)]. Such a distribution of operators can be explained by the start molecule being more complex than the target one. The breadth of the chemical space explored is a function of the morphing operators available. Currently, unless decoy molecules are used, these operators generate local chemical subspace. Coarser operators (e.g., add/remove ring) would generate chemical space containing more chemotypes, and we are considering expanding the set of morphing operators in future versions of the software.

Indeed, Molpher is undergoing continuous development, with several other new features planned for future releases. These include an enhanced graphical user interface, an improved Bayesian synthetic feasibility filter, new visualization methods, the inclusion of various drug-like, lead-like and unwanted substructure filters [75], and the possibility of generating morphs containing only user-defined substructures. To facilitate predictive compound design in a better way than it is currently possible via directed structural modifications, we also plan to incorporate biological activities and ADME/Tox properties into the morphing process. Furthermore, the algorithm will be modified to implement the multiobjective optimization approach [76], which will enable the morphing process to be driven by several properties (high activity, low toxicity, etc.) simultaneously.

## Conclusions

We have described a molecular morphing tool, Molpher, which, to the best of our knowledge, is the first freely available implementation of the concept also known as ‘chemical space travel’ [46]. Molecular morphing is a computational strategy for the systematic exploration of chemical space. Given a start/target molecule pair, the algorithm iteratively produces a path covering a structural continuum between them. This is done by the iterative application of simple structural changes, such as adding or removing an atom or bond. Molpher can be used via a fully-fledged desktop application or run in batch mode. Molpher’s modular architecture guarantees easy extensibility, thereby enabling the simple adoption of new features in the future. Our results show that Molpher is capable of rapidly finding a path in chemical space, even for relatively distant molecules. The molecules forming the resulting path are restricted to subspace laid out by the start/target molecule pair. Restricting the explored chemical space to those compounds amenable to chemical synthesis does not lead to a significant increase in computational demands.

In our opinion, the compounds encountered on and around a morphing path could provide valuable starting points for future *in silico* or *in vitro* experiments aimed at assessing the biological activities of such compounds. We believe that Molpher is a useful software component that could be easily incorporated into any existing computational drug design pipeline.

### Availability and requirements

Project name: Molpher.

Project home page: http://siret.cz/molpher/.

Operating system(s): MS Windows (client and server), Linux (server).

Programming language: C++.

Other requirements: MS Windows client and server are provided both as pre-compiled binaries, as well as C++ source code. Linux server is provided as C++ source code only. Precompiled binaries have no other requirements. To compile Molpher, several dependencies must be satisfied. These are listed in readme files in the source code distribution.

License: GNU General Public License Version 3, 29 June 2007.

Any restrictions to use by non-academics: None.

## Competing interests

The authors declare that they have no competing interests.

## Authors’ contributions

DS instigated the project, proposed the molecular morphing approach, participated in the development of the software, collected data sets, and drafted the manuscript. DH is the lead developer of Molpher. He, together with DS, proposed the molecular morphing approach, designed and implemented the algorithm and the graphical user interface, and helped to draft the manuscript. PŠ developed and implemented visualization methods, and he maintains the code base. MV implemented the synthetic feasibility algorithm. All authors read and approved the final manuscript.

## Supplementary Material

Additional file 1 A detailed description of the molecular morphing algorithm. Click here for file

Additional file 2 A list of molecular fingerprints and molecular similarity coefficients available in Molpher. Click here for file

Additional file 3 ***D1*, *D2*, and *D3* datasets. **Each line consists of SMILES representation of the structure and its Pubchem ID. Each odd line contains the start structure, its corresponding target structure is given in the next line. Click here for file

Additional file 4 Examples of paths produced by Molpher. Click here for file
