# Supplementary material for: Molpher: a software framework for systematic chemical space exploration
Source: J Cheminform. 2014 Mar 21;6:7. doi: 10.1186/1758-2946-6-7 (PMC3998053; doi:10.1186/1758-2946-6-7)
Supplement: Additional file 1 — A detailed description of the molecular morphing algorithm. [file 1758-2946-6-7-S1.pdf]

1 Algorithm description

A molecular morphing's pseudocode is shown in Algorithm 1. Molpher builds a so-called exploration tree that stores all molecules encountered during the exploration process. At the beginning the tree contains only one leaf - the source molecule M_S. At each step i all molecules in the leafs of the tree (we refer to them as"morphs") form a set $\mathcal{M}_i$. A new set of candidate morphs is generated from $\mathcal{M}_i$ by an application of randomly chosen morphing operators over each of the molecules in $\mathcal{M}_i$. Each morph must comply with basic chemistry rules meaning that every its atom must have the correct valency. The set $\mathcal{M}_{i+1}$ is union of molecules from the candidate set which are accepted and molecules from $\mathcal{M}_i$ which do not produce an offspring, i.e. none of their candidates get through int $\mathcal{M}_i$. To choose which candidates to accept, following procedure takes place. The candidates are ordered according to their distance from the target molecule M_T and their position determines whether they will be accepted. Concretely, given number of the closest candidates are accepted (parameter `cnt_accept`) and the remaining are accepted with a probability proportional to their distances (procedure *Accept* in the code). In total, the number of candidates is dictated by the parameter `cnt_accept_max`. Obviously, not every leaf from $\mathcal{M}_i$ has an offspring in $\mathcal{M}_{i+1}$. If a leaf does not produce an offspring during several iterations (given by the `cnt_it_prune` parameter, see below) it is discarded from the exploration process (see below the pruning description). The exploration process terminates when the target molecule is found in the $\mathcal{M}_{i+1}$ set. The sequence of morphs between M_S and M_T defines the resulting path $\mathcal{P}$.

The morphs are generated using the procedure *GenerateMorphs*. To increase a chance of reaching a target structure its vicinity is explored in a more detail. In early stages when resulting morphs are located far (given by `dist_det` parameter) from the target molecule `cnt_morphs` is generated. When closer to the target, higher number of morphs `cnt_morphs_det` is produced.

Morphs with undesired features may be discarded from the exploration process utilizing the *ApplyFilters* function. Currently, two filters are implemented in Molpher. The synthetic tractability filter [1] removes molecules which can not be easily synthesised, and the molecular weight filter prevents the formation of too large molecules that do not posses drug-like properties [2].

Similarly to optimization problems also the exploration process may get stuck in a local minimum, i.e. in the subspace containing structures close to the target surrounded by molecules with larger distances to the target. To avoid this problem we implemented a pruning procedure *Prune* accepting two

Algorithm 1 Molpher's algorithm *(Computes path P between source molecule M_S and target molecule M_T)*

$\mathcal{M}_0 \leftarrow \{M_S\}$
$P \leftarrow \emptyset$
for $i = 1$ to `params.cnt_max_iterations` **do**
  $\mathcal{M}_i \leftarrow \emptyset$
  for all $M \in \mathcal{M}_{i-1}$ **do**
      /* *Generate* ***`params.cnt_morphs`*** *or* ***`params.cnt_morphs_det`*** *morphs from M depending on the distance of M to M_T and the value of* ***`params.dist_det`***. */
    $\mathcal{M}_{aux} \leftarrow$ GenerateMorphs(M, `params.cnt_morphs`, `params.cnt_morphs_det`, `params.dist_det`)
    $\mathcal{M}_{aux} \leftarrow$ ApplyFilters($\mathcal{M}_{aux}$)
      /* *Application of filters such as synthetizability filter, molecular weight filter, ...* */
    for all $M_{aux} \in \mathcal{M}_{aux}$ **do**
      M_{aux}.pred $\leftarrow M$
    end for
    $\mathcal{M}_i \leftarrow \mathcal{M}_i \cup \mathcal{M}_{aux}$
  end for
  if $\exists\ M \in \mathcal{M}_i$: IsIdentical(M, M_T) **then**
      /* *Generate the path based on the M.pred information* */
    P = GeneratePath(M)
    break
  end if
  Sort($\mathcal{M}_i$, δ, M_T)
      /* *Accept only* ***`params.cnt_accept`*** *morphs from $\mathcal{M}_i$ being most similar to M_T. The rest of the molecules will be accepted with a probability based on their position in the sorted list.* */
  $\mathcal{M}_i \leftarrow$ Accept($\mathcal{M}_i$, `params.cnt_accept`)
      /* *Only* ***`params.cnt_accept_max`*** *morphs can be accepted.* */
  $\mathcal{M}_i \leftarrow$ CutOff($\mathcal{M}_i$, `params.cnt_accept_max`)
      /* *Remove those molecules M (and all their successors) having no successor closer to M_T than M for* ***`params.cnt_it_prune`*** *number of iterations. If* ***`params.cnt_morphs_max`*** *directions have been explored for a given molecule, remove it from the exploration process.* */
  Prune(`params.cnt_it_prune`, `params.cnt_morphs_max`, $\mathcal{M}_1, \ldots, \mathcal{M}_i$)
end for
return P

parameters: `cnt_it_prune` and `cnt_morphs_max`. If the number of iterations, during which no morph closer to the target occurs, reaches `cnt_it_prune` the molecule and its subtree is removed from the exploration tree. Pruned molecules are remembered, and they can not be explored again in the future. To avoid a repetitive exploration of non-promising areas of the chemical space we control the number of morphs generated from a particular molecule. If this number exceeds `cnt_morphs_max` the molecule is removed from the exploration process, and no other future path is allowed to contain this molecule.

2 Algorithm parameters

Molecular morphing algorithm is influenced by several user-defined parameters. Their default values used in Molpher are given in parenthesis.

- `cnt_max_iterations` (1000) – Upper limit of the number of iterations. If a path is not identified within `cnt_max_iterations` the algorithm terminates.

- `cnt_morphs` (90), `cnt_morphs_det` (200), `dist_det` (0.1) – Number of morphs generated from one molecule in one iteration. Higher number of morphs requires more time but better solution may be identified than with lower number of morphs. If the molecule's distance from the target is lower than `dist_det`, `cnt_morphs` morphs is generated. After reaching space closer to the target the morphing process becomes more detailed, and `cnt_morphs_det` is generated from each molecule.

- `cnt_accept` (40), `cnt_accept_max` (150) – Number of morphs that will be accepted into the next iteration. All morphs in the candidate set are sorted in an ascending order by their distance from the target molecule. First `cnt_accept` morphs are always chosen for the next iteration. Then each of the rest of morphs is accepted with a probability proportional to its distance from the target. The algorithm checks morphs one by one until remaining `cnt_accept_max-cnt_accept` candidates are selected. The `cnt_accept` and `cnt_accept_max` parameters represent a trade-off between time/space constraints and quality. By storing a larger set of candidates we increase both memory requirements and runtime. However, a higher number of candidates allows us to explore larger area of the chemical space thus increasing a probability of finding a solution.

- `cnt_it_prune` (6) – Number of iterations after which a molecule and all its morphs are discarded. This parameter prevents the algorithm to get

stuck in the local minima. If set too low the probability of discarding an useful solution is increased. If set too high the runtime is increased.

- `cnt_morphs_max` (5000) – Maximum number of morphs that can be generated from a single molecule. This is a global parameter controlling the same molecule occurring in various iterations. Decreasing this parameters decreases the runtime but increases a chance of missing a path.

- `cnt_weight_min` (0 Da), `cnt_weight_max` (500 Da) – Minimum and maximum molecular weight (in Daltons) of morphs.

References

[1] Peter Ertl and Ansgar Schuffenhauer. Estimation of synthetic accessibility score of drug-like molecules based on molecular complexity and fragment contributions. *J. Cheminformatics*, 1:8, 2009.

[2] C. A. Lipinski, F. Lombardo, B. W. Dominy, and P. J. Feeney. Experimental and computational approaches to estimate solubility and permeability in drug discovery and development settings. *Advanced drug delivery reviews*, 46(1-3):3–26, March 2001.
