# Supplementary material for: Molpher: a software framework for systematic chemical space exploration
Source: J Cheminform. 2014 Mar 21;6:7. doi: 10.1186/1758-2946-6-7 (PMC3998053; doi:10.1186/1758-2946-6-7)
Supplement: Additional file 2 — A list of molecular fingerprints and molecular similarity coefficients available in Molpher. [file 1758-2946-6-7-S2.docx]

# List of fingerprints and similarity coefficients implemented in Molpher

Fingerprints and molecular similarity coefficients in Molpher are computed using RDKit functions. Available options are described in RDKit documentation [1]. FP(ext) is a fingerprint's extended version that augments FP with a set of additional molecular characteristics such as, e.g., the number of atoms of each atom type.

## Fingerprints

- Atom pairs
- Morgan
- Topological
- Topological layered 1
- Topologocal layered 2
- Topological torsion
- Atom pairs (ext)
- Morgan (ext)
- Topological (ext)
- Topological layered 1 (ext)
- Topologocal layered 2 (ext)
- Topological torsion (ext)

## Similarity coefficients

- All bit
- Assymetric
- Braun-Blanquet
- Cosine
- Dice
- Kulczynski
- McConnaughey
- On Bit
- Russel
- Sokal
- Tanimoto
- Tversky (substructure)
- Tversky (superstructure)

# References

[1] http://www.rdkit.org/docs/GettingStartedInPython.html#fingerprinting-and-molecular-similarity
