# Supplementary material for: Molpher: a software framework for systematic chemical space exploration
Source: J Cheminform. 2014 Mar 21;6:7. doi: 10.1186/1758-2946-6-7 (PMC3998053; doi:10.1186/1758-2946-6-7)

## Examples of paths produced by Molpher

Molecular morphing is a non-deterministic algorithm. If run several times it produces different paths. Five paths between pentamidine (CID 4735) and 2-imino-3-(1H-indol-3-yl)propanoic acid (CID 5599)

molecules from the *D3* set (30-40% similarity between the start and the target structures) demonstrate this feature.

### Path 1


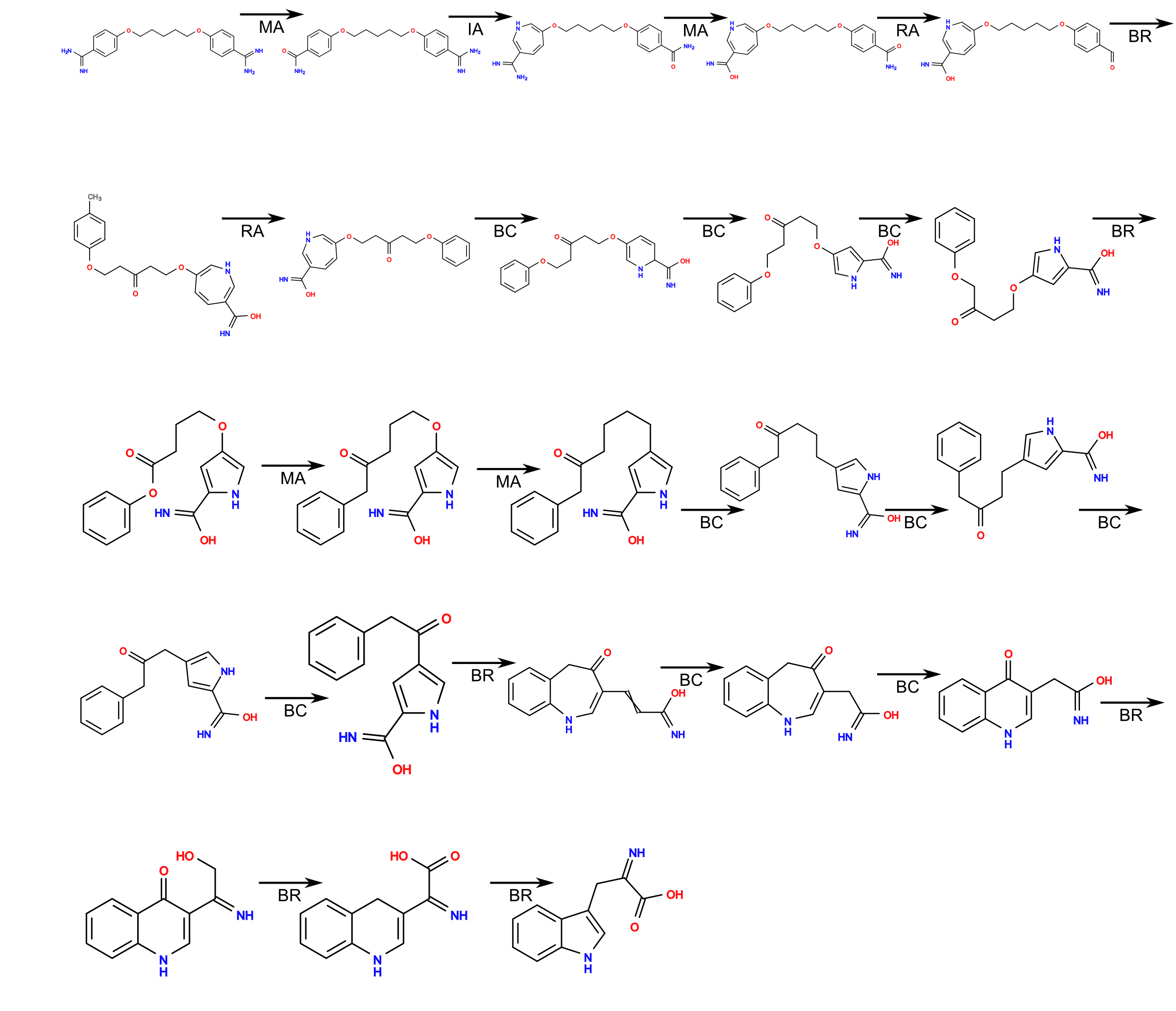


### Path 2


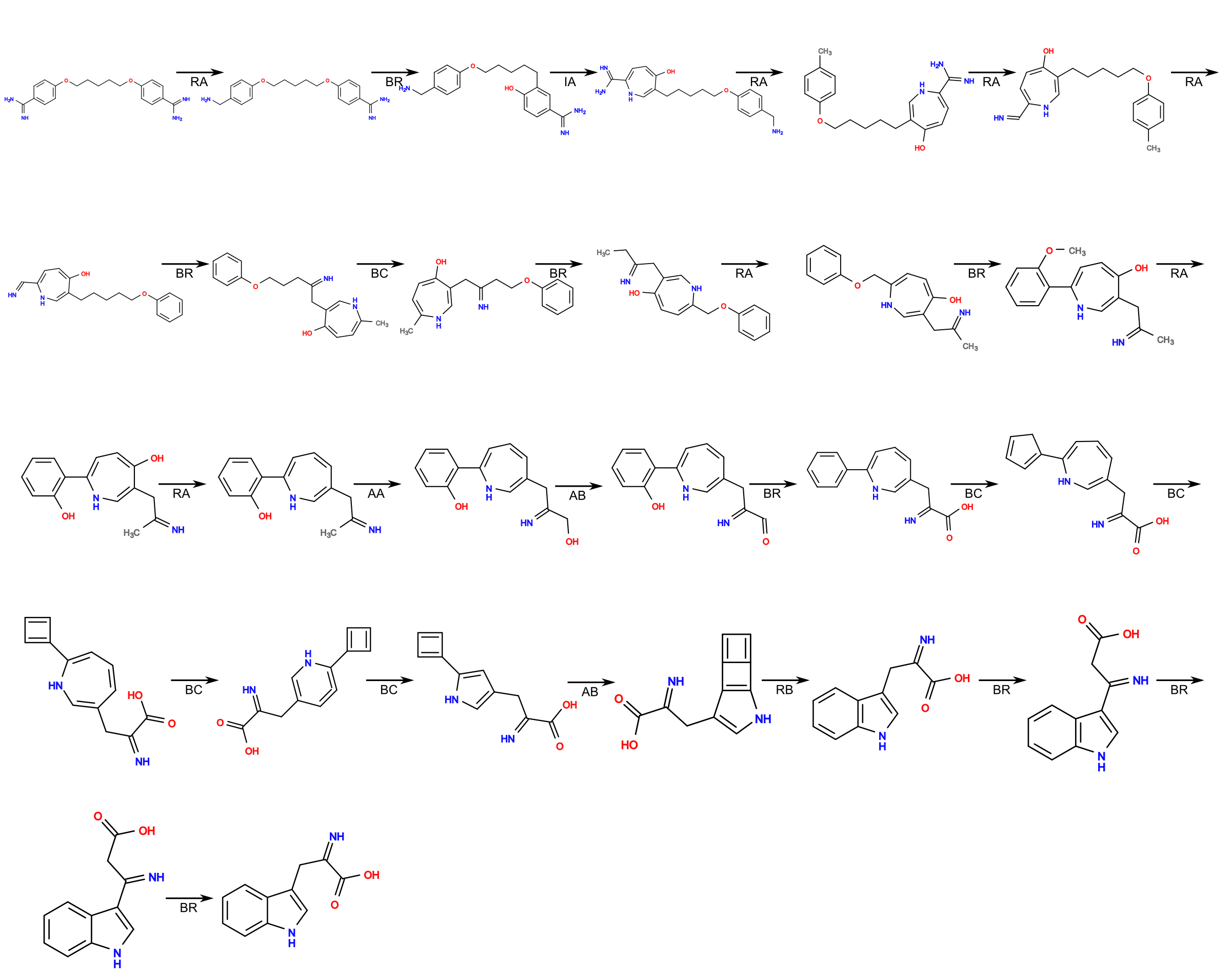


### Path 3


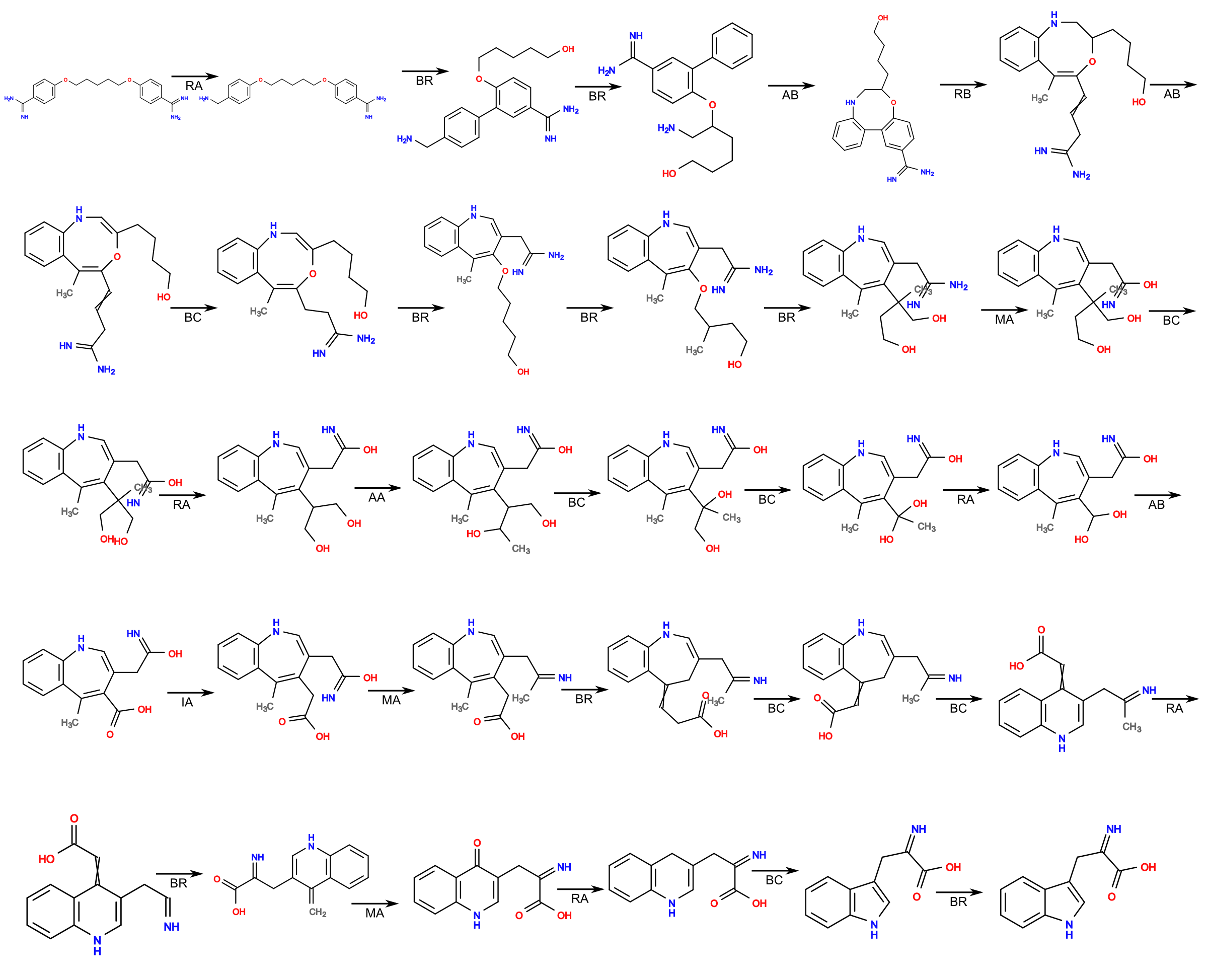


### Path 4


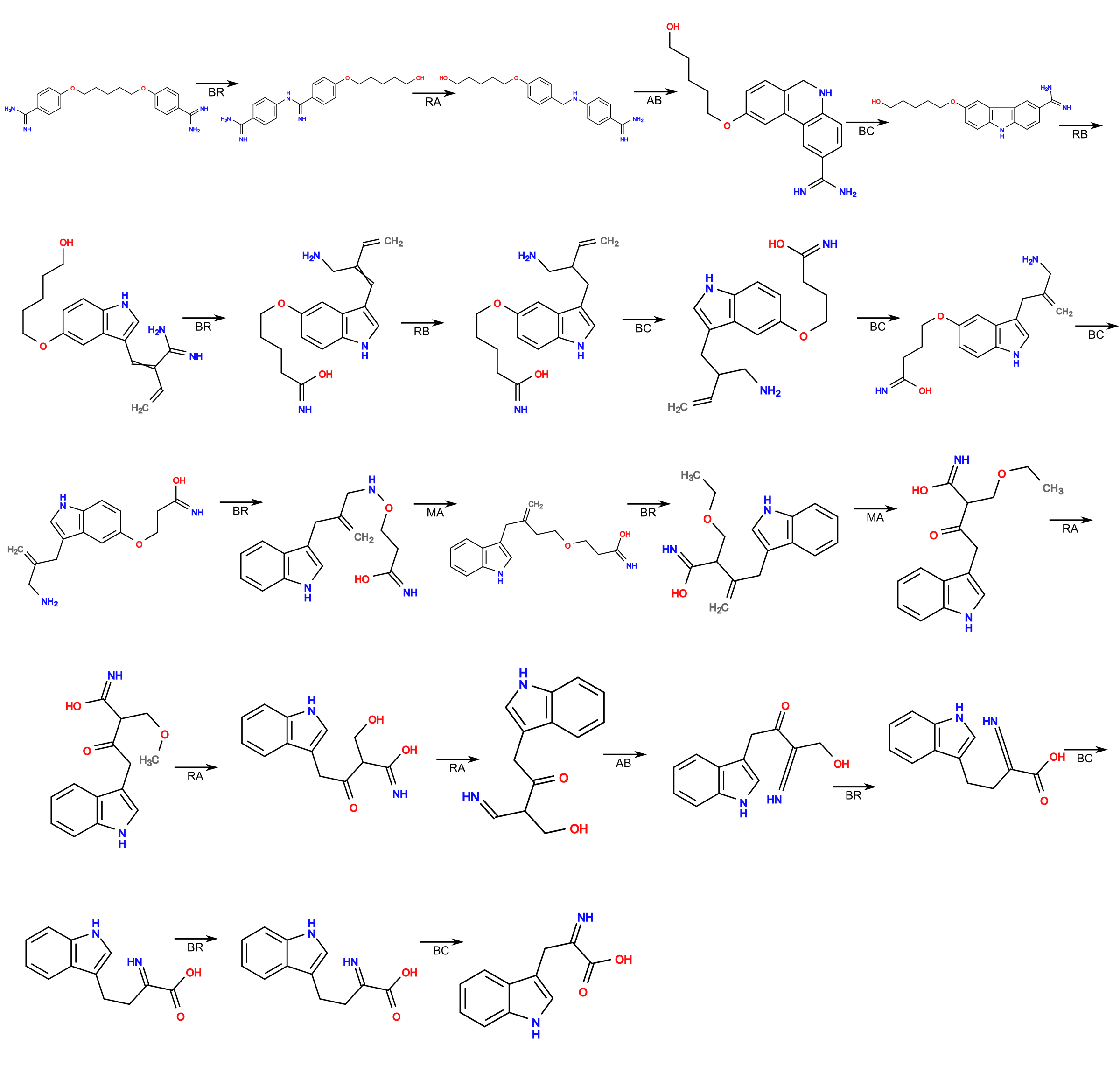


### Path 5


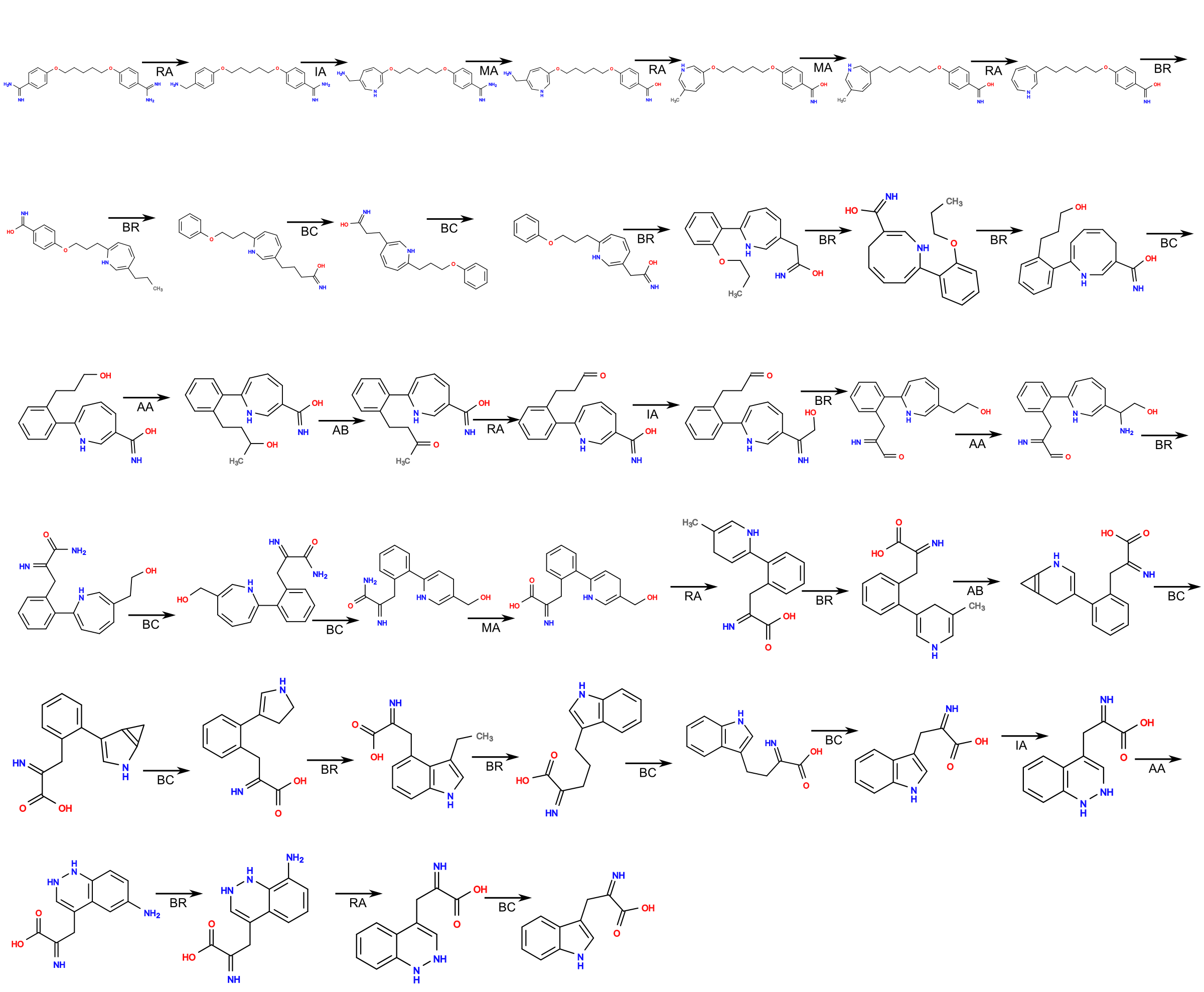

Supplement: Additional file 4 — Examples of paths produced by Molpher. [file 1758-2946-6-7-S4.docx]
